# Our right to in vitro fertilisation—its scope and limits

**DOI:** 10.1136/jme.2008.024232

**Published:** 2008-10-24

**Authors:** T Tännsjö

## Abstract

There exists a derived negative right to procreative freedom, including a right to in vitro fertilisation (IVF) and to the exercise of selective techniques such as preimplantation genetic diagnosis. This is an extensive freedom, including not only the right to the exercise of a responsible parenthood, but also, in rare cases, to wrong decisions. It includes also a right for less than perfect parents to the use of IVF, and for IVF doctors to assist them, if they want and can agree about the terms.

Do we have a right to assisted reproduction? In particular, do we have a right to in vitro fertilisation (IVF) treatment, irrespective of whether we suffer from infertility? Yes, we do. In this paper I will state the rationale behind this claim and also indicate the scope and limits of it. In order to do this, I will need to make some preliminary distinctions. What does it mean to have a right in the first place? I will distinguish between a view where rights are seen as fundamental and a view where they are seen as derived. I will defend the view that they should be conceived of as derived. I will also distinguish between positive and negative rights. In the present context I will focus exclusively on negative rights. After having made these preliminary distinctions I will defend my claims and delineate the rough contours of the scope and limits of our right to IVF treatment.

## BASIC VERSUS DERIVED RIGHTS

Some moral philosophers see morality as basically constituted by rights. Their point of departure is the idea that certain creatures, moral agents or persons, own themselves. This is an idea of a moral self-ownership, which implies that moral agents have a basic moral right to dispose with themselves as they see fit. They have a right to themselves. This right to self-ownership is a basic moral notion. There are obligations as well, according to this view of morality, but these are derived from the basic rights. You may do to yourself whatever you like, but I am not allowed intentionally and actively to interfere with you. I ought to abstain from doing so. You may kill yourself, if you like, and you may hire me to kill you, and I am then allowed to kill you, but I am not allowed to kill you against your wish. We may sum up this idea thus: everyone is free to do as she sees fit with herself, everyone is free to exercise her right to self-ownership, but only to the extent that this does not interfere with a similar right of everyone else.

Given this notion of a moral right we may sometimes, when we state that it is wrong to do a certain thing against a certain individual, come to back up this claim with the moral claim that, to do this against this individual, would be to violate this individual’s basic right to self-ownership. The idea of a moral right then appears in the premise of a moral argument.

In the present context I will have none of this. I do not believe that basic rights of this kind exist. There are both moral and methodological problems with the claim that they do exist that seem to me to be insurmountable. The moral problems have to do with the fact that, by respecting a putative basic right to self-ownership, we may come to make the world a much worse place. We may even come to encounter situations where the number and seriousness of rights-violations increase, just because we hesitate to violate an individual right. This is not acceptable from a moral point of view. Furthermore, the moral rights theorists are at a loss when we invite them to explain more exactly what it means to be a moral agent, a person, a rights bearer. And even if they were able, in the final analysis, to answer this question in a satisfactory manner (we would at least understand what they are referring to), they would still be at a loss when we ask them to explain why, from a moral point of view, moral agents or persons are so special. Why are they more important, in particular, from a moral point of view, than other creatures capable of feeling pain and pleasure? Lack of personhood may be a good reason not to hold someone responsible for her actions, but hardly a reason not to grant her rights; that is, lack of personhood is hardly a reason not to take the interests of someone into moral consideration.

In the present context these observations must suffice to set to one side the idea of moral rights as basic. The important thing to note here is that, even if we do not believe in basic moral rights of this kind, there is yet plenty of room for moral rights talk in our moral discourse. We may think of moral rights as derived from basic consequentialist (utilitarian) considerations. We may think of them as *legal* claims that society *ought* (morally speaking) to grant people. This is, for example, what J S Mill has to say about rights:

When we call anything a person’s right, we mean that he has a valid claim on society to protect him in the possession of it, either by the force of law, or by that of education and opinion ... To have a right, then, is, I conceive, to have something which society ought to defend me in the possession of. If the objector goes on to ask, why it ought? I can give him no other reason than general utility.^1^

This Millean view of rights is the one I am going to adopt in this paper. I elaborate on it elsewhere.^2^ Note that on his view, the rights talk appears in the conclusion of a moral argument rather than among the premises of it.

## POSITIVE VERSUS NEGATIVE RIGHTS

When rights are conceived of as basic it is common to see them as merely negative. I have a negative right against everyone else to exercise my right to self-ownership in the sense that no one should actively and intentionally stop me from doing so, unless by exercising my right I violate the right of anyone else. By conceiving of them as negative we can ascertain that they do not conflict. There is always of way of respecting all the rights that exist. However, when rights are conceived of as derived it is natural to make a distinction between positive and negative rights. Negative rights can be conceived of in the manner already indicated. I have a negative right to something such as IVF treatment if society should see to it that no one actively and intentionally stops me from accessing IVF treatment, if I seek such treatment. And those who are willing to provide me with this treatment, on the terms we have agreed upon, should be allowed to do so. To have a positive right to IVF treatment means that I have a negative right to it, but also something more. It means that, not only should no one stop me from accessing IVF treatment if I seek such treatment, I should also be provided with such treatment, if I want it. For example, if such treatment, when offered on the market, is too expensive for me, then my use of it, if I want it, should somehow be subsidised.

I believe we also have a positive right to IVF treatment hence explained, but this is not the subject of this paper. I have argued this point elsewhere.^3^ Here I will focus on negative rights and try to establish not only that such a negative right to the use of IVF exists, but also that its scope is more far-reaching than has sometimes been assumed.

A problem with positive rights is that they can conflict. A further problem is that it is not always clear who has the obligation to see to it that positive rights are fulfilled. These problems are not insurmountable but they are genuine. It is hence a nice aspect of negative rights that they can be devised such that they do not conflict. There is always a way for everyone to act such that no negative rights are being violated. As was pointed out above, this is how those who conceive of negative rights as basic, usually conceive of them. It is equally natural, however, for those who conceive of negative rights as derived from consequentialist considerations to see to it that they do not conflict. Legislation in general, and legislation granting negative rights of various kinds in particular, should be free of practical inconsistencies. This means that when I claim that we have a negative right to IVF treatment it is crucial for me to see to it that the regulation of this right is devised in a manner that does not mean that, when exercised, it comes to violate important rights and interests of others. Let us now see how all this applies to IVF.

## A NEGATIVE RIGHT TO IVF TREATMENT

The ordinary purpose of an IVF treatment is to see to it that a healthy and welcome baby is born. Even if, in our somewhat perverted culture, killing, destruction and violent behaviour seem to be held in high esteem and the rather mundane procreative actions are rarely celebrated (at least if we set aside the sexual act as such), these actions are good actions (in general). Most people have children. In our ordinary lives, our procreative actions are the best actions we ever perform. When we are at our jobs, we can rarely, most of us, feel confident that we make the world a better place. There is an exception from this rule, of course. Those who work at IVF clinics are pure do-gooders! Even if, otherwise, in our lives, we who are not working at IVF clinics make little difference to the sum-total of wellbeing in the universe, by having children we probably in most cases make a difference, however, and a difference for the better. This counts heavily in support of the claim that it is all right to conceive and parent children. I would even go so far as God does in Genesis, when he claims that we *ought* to replenish the world. I have consistently argued for this claim over the years,^4^ ^5^ claiming that what Derek Parfit^6^ has nick-named the “repugnant” conclusion is a misnomer. Be that as it may, in most cases, when we have children, it is *right* to do so. This seems to be accepted by Parfit in his “mere addition paradox”,^6^ where he takes for granted that by merely adding happy people to the world we do not make the world a worse place. I assume this means that it is all right to add happy people to the world. But then society should at least grant us a negative right to procreate.

Now, some people have fertility problems. They need to have access to IVF in order to be able to have children. Society should not stop them from doing so, since having children is, in most cases, a good deed, this is true no less of people who use IVF. If there is a difference, it probably counts in favour of those dedicated parents who conceive with the aid of IVF.

This should suffice to grant that we have derived a negative right to procreation in general and to the use, if necessary, of IVF in particular. And if such a negative right exists, that is, if society ought to grant it through suitable legislation, we can also deduce as a corollary the right for anyone who wants to assist people who seek IVF to do so. No one should be stopped from either asking for IVF treatment or from providing such treatment, on terms acceptable to both parties.

But how far should the negative right to IVF treatment be extended? Are there crucial other interests involved, that might provide a rationale for restricting somehow the right?

One may come to think here of the following interests: (i) the interest of the prospective child, (ii) the interest of the world (considering that negative rights are derived from consequentialist considerations, and (iii) interests of people who are exposed to prejudice in society (in particular, to disabled people).

I will discuss these three possibilities in order.

## THE INTERESTS OF THE PROSPECTIVE CHILD

I have argued that, by conceiving and parenting children, in ordinary circumstances, we make the world a better place. The worst sin, the ultimate sin, I am prepared to add, would be if we all abstained from having children, hence putting an end to humanity. But even if this is true there are exceptional cases, whereby having children we make the world a worse rather than a better place. This can have something to do either with problems pertaining to the children themselves, or to do with our capacity or willingness for parenting. Are there not cases where the child would be born to such a bad life that, in the interest of the child, the access to IVF treatment should be denied?

This argument presupposes that we can make predictions about the future of the child. It is very difficult in many cases to make such predictions. However, in some cases the predictions may seem easy to make, so let us focus on those cases. Should at least the access to IVF be denied in these cases?

Let us first consider cases where we know that the prospective child runs a risk of being born to a terrible life due to illness or handicap. Are there such cases? Yes, admittedly, there exist a few such cases. I think of cases where a child is born with a condition such as Krabbe’s disease. The child is destined, because of its predictable genetic condition, to a short and painful life. It is correct, in such cases to claim that, if such a child is born, it would have been better for it never to exist. By conceiving such a child we wrong the child, I am prepared to say. Some have denied this claim on the ground that it makes no sense to compare existence to non-existence—see for example David Heyd about this.^7^ But we need not claim that there is a fact of the matter as to how a not existing child would have fared in order for the claim to make sense that an existing individual would have been better off had he not existed. The claim merely amounts to saying that life is bad for this child and a world where this child, with her suffering, is missing, is in this respect better than the existing world. And it is an existing child who experiences the pain, that makes the difference between these two worlds.

Does this mean that, if a couple runs the risk (25%) of having a child with Krabbe’s disease, they should not be provided with IVF?

This seems to be the wrong conclusion. We should rather conclude that, if such a couple wants to conceive a child through IVF, we should also offer them pre-implantation genetic diagnosis (PGD). Then they can have a healthy child without risking having a child with the terrible disease.

Does this mean that, if a couple who runs the risk of having a child with Krabbe’s disease refuses to use PGD when conceiving a child, they are acting irresponsibly?

It does indeed, or so I am prepared to argue. Does this also mean that the use of PGD should be compulsory for the couple? Otherwise IVF treatment will not be provided?

There are good reasons to draw this conclusion but, as we will see, there are even stronger reasons against drawing it. I will have to postpone the discussion about this until I consider the interests of disabled people in relation to IVF and PGD treatments.

Thus far we have just concluded that there are cases where it is morally wrong to conceive children, when the matter is assessed from the point of view of the prospective child. It is wrong to conceive a child who is destined to a terrible life. However, these cases are very rare. It is not plausible to claim, for example, that a life with, say, Huntington chorea, is another example of a life not worth living. A life with Huntington chorea is a life with less happy life expectancy than a life without the disease, but this does not mean that such a life qualifies as a life not worth living. It is a life less worth living than many lives—as a rough estimate we may claim that it contains only half the sum-total of wellbeing characteristic of an ordinary life—but yet, absolutely speaking, it could very well be a good life.

But should not children be provided with something more than a life just worth living? Are we not under an obligation to see to it that the child as a chance to live an ordinary life, a life beyond a reasonable “critical level”? Is it not to wrong a child to put it into existence, if its existence is below this level—even if the child, when asked, is still pleased to be around?

This is a popular idea both in applied ethics^8^ and population ethics proper,^9^ ^i^ but yet a false one. It leads to what Gustaf Arrhenius has called the sadistic conclusion.^13^ If it is morally bad to create a happy individual, being just below the putative critical level, then we must conclude that it is better to have a world with one person who suffers terribly (who is well below the line where life begins to be worth experiencing), than a world with very many people leading lives worth living but living just below the critical level. This is absurd. There are few knock-down arguments in philosophy. This is one of them.

All this means that parents should be granted the use of IVF, even when they run the risk of having children with terrible diseases. If they do, they should also be offered PGD. This means that we allow them to conceive children in a responsible manner. I leave it for a separate section to discuss whether they should also be required to use PGD, in order to obtain IVF treatment, when facing risks such as these.

Now, if prospective parents, who run a risk of conceiving children with terrible diseases, should be offered PGD, when, because of fertility problems, they need to access IVF, it is also very natural to extend their right to conceive children in a responsible manner to a right to IVF treatment, even when they have no fertility problem. They should be allowed to procreate with the use of IVF in order to procreate in a responsible manner. Of course, ordinary procreation and prenatal genetic testing is also an alternative for them, but if they do not want to risk several abortions, it may be preferable for them to seek IVF and PGD. If they do, no one should stop them from doing so.

Let us now focus on the prospective parents as such. Could one make a case for the claim that the prospective parents are not good enough to deserve the right to treatment? Having seen that, in order to refuse treatment with respect to the right of the prospective child, we must conclude that it is difficult to defend this claim. In order to do so it is necessary to show that the life of the child, if it is allowed to come into existence, is not worth living. This is rarely the case, even with poor parents.

Poor parents may mean that a child has a worse life than it would have had with better parents, but this is no option for the IVF child. If we refuse to provide treatment to the couple because we believe that they will become poor parents, this does not mean that the same child will be born with better parents. It means that it will not be born at all. And in most cases, even when parenting is less than perfect, children are capable of developing normally and eventually they will have lives well worth living. And in the few cases where the parents are totally incapable of parenting, society takes over and takes custody of the child, who is provided either with foster parents or, in some cases, is given away for adoption.

It is true that some children who have been placed in foster homes or who have been given away for adoption lead lives not worth living. Some of them kill themselves. However, this is the exception. Most children in these categories live lives worth living. It is impossible to predict who will be among the rare cases where life will not be worth living. So we cannot deny IVF treatment on the ground that the parents are not good enough. Provided they are capable of following necessary medical advice and to go through with the treatment, they have a right to do so. At least there is no reason to deny them this right on the ground that it is not in the best interest of their prospective children to be born. The rationale behind this right is the same as the rationale behind the right to conception in general, with our without IVF. In most cases, conception results in an additional happy life.

A comparison with adoption is here instructive. If a child has been abandoned and is in need of parents who can adopt it, it makes sense to claim that not just any couple is good enough. We are here speaking of a child with special needs. It makes sense to claim that only the best is good enough for this child. The situation when a couple requests IVF services is different. They have no child, yet. If they are denied the service, their prospective child will never be born. Here we cannot say that only the best is good enough for the child, with respect to parents. There are no putative alternative parents to compare with. However, if it turns out that an IVF child, because its parents do not take proper care of it, needs to be given away for adoption, then, of course, only the best is good enough for this child.

## FROM THE POINT OF VIEW OF THE WORLD

We have seen that, if a child is born to a life worth living, then, when it was allowed to be conceived, no rights possessed by it have been violated. However, in some cases the prospective parents face a choice. Either they conceive a child and run the risk of conceiving a child with a disease that destines the child to half a life rather than a full life, such as when the child is inflicted with Huntington chorea. Now, if parents want to conceive through IVF, even though they run a 50% risk of conceiving a child with Huntington chorea, should they be allowed to do so?

We have seen that, even if they conceive, and even if it transpires that the child they have conceived has Huntington, there is no reasonable complaint that could be made by their child. It has a life worth living. Its life would have been better for it, if it had not been inflicted with this disease, but this was no option for this child. So no right of this child has been violated when it was conceived. However, if the parents have an option, either to conceive a child with Huntington, or a child without Huntington, are they not under a moral obligation to conceive a child without Huntington? And is this not perfectly possible for them to do, if they resort to PGD?

I think parents who run a risk of having a child with a disease, which lowers the expected value of the child’s life, have a moral obligation to select a healthy child instead of the child they risk to have. They are under a moral obligation to use PGD, then. But how can this obligation be defended?

Obviously, it cannot be defended with the claim that, if they have a healthy child rather than a sick child, there is someone for whom this would be better. And still they have such an obligation, I believe. And this has to do with the fact that, if they have the healthy child, the world as such will be better. When conceiving children, we ought to adopt a God’s eye view of our choice. We ought to see it as a choice between possible worlds, and we ought to opt for the best possible one among those who are available to us.

Now, this conclusion cannot come as any surprise to any utilitarian. This is what utilitarianism requires from *us all the time.* And if rights are derived from consequentialist considerations, it might then be tempting to argue, also, that not only should parents who want to exercise their procreative rights in a responsible manner be allowed to use PGD, but also that, unless they do so, they should be denied access to IVF in the first place. If they are not prepared to select against Huntington chorea, then they should be denied access to IVF. However, there are good reasons to resist this temptation, and in the final section of this paper, to which I now turn, I will explain why.

## DOES THE EXERCISING OF REPRODUCTIVE CHOICES POSE A THREAT TO DISABLED PEOPLE?

I have argued that, not only are we under a moral obligation to avoid, if possible, to conceive children who will live terrible lives, lives not worth living, but that, furthermore, when we have a choice, we ought to opt for those children, among all possible children we can conceive, that will have the best chances to lead a good life. All this means that it makes sense, not only to use IVF when necessary, because we suffer from fertility problems, but also to use it when we don’t, in order to be able to use methods of selection such as PGD. But does not the use of our right to choose children mean that the lives of people living with various different disabilities are put at risk? Or, does it not at least mean a threat against their self-esteem? In particular, does it not mean that prejudice against people living with disabilities will spread?

This concern is a serious and a genuine one. It should be taken seriously. However, and alas, there is no ideal way of handling it. Of course, if we could prohibit all kinds of use of techniques rendering a selection of children to be born possible, we may have obviated the threat felt by people living with diseases and conditions selected against. However, this is neither a reasonable nor a feasible strategy. It is unreasonable because it requires, in rare cases, that responsible putative parents are not allowed to behave in a moral manner; instead they are forced into risking the birth of children who are destined to lives not worth living. And it is not feasible since these techniques are already with us, and a prohibition in one country would only lead to medical tourism. How then, can we best handle the concern, if not by prohibiting the selective techniques? Should we regulate their use or should we allow that it be used as prospective parents see fit? These seem to be the two remaining options.

It is tempting to argue that, while some choices should be allowed, others should be prohibited. Prospective parents should not be allowed to choose the sex of their children, for example, let alone, if it becomes possible, should they be allowed to choose sexual orientation of their children. Why? Well, I suppose the argument must be that it is no better to be born with one sex rather than the other, or with one sexual orientation, rather than the other.

However, if this is how society regulates the use of the selection techniques society sends out a message. Partly, this message is fine: there is no problem being female or gay. However, when, at the same time, society allows for other reproductive choices, such as a choice against a child with a mental handicap (Down syndrome, say), it does send out a rather nasty message. Down syndrome is indeed a problem! This means that the Nazi spectre is once again alive.

So in order to avoid a situation where society has a view on what kind of lives are worth living, we should allow the prospective parents to exercise a complete freedom in this respect. This means that, while some parents find, for example, that a deaf child is just too much right now (they are not prepared to migrate into another culture, which is alien to them, they are not prepared to learn a new language, and so forth), these parents are allowed to make a selection against deafness; at the same time, another (deaf) couple may welcome a deaf child; they are even free to make a selection in favour of deafness.

Does this mean that many children will be born because of immoral choices made by their parents? Does it mean that some children will be born to lives not worth living? Will it mean that some parents deliberately conceive children with less chances of having full and happy lives than children with better chances?

To some extent, this is bound to happen. However, in most cases prospective parents are very eager to see to their prospective children’s best interest. So in most cases, procreative freedom and a freedom to select will have good consequences. And there is no guarantee that society will be successful, if, instead, it takes over responsibility for these choices. And when society goes wrong, it may go wrong on a large and terrifying scale. Moreover, even if society (the state, the doctors, the medical authorities, or what have you) were more successful than most prospective parents in making these decisions, which I very much doubt that it would be, the very fact that we had endowed society with the right to decide what sort of people there should be would mean a serious threat to disabled people. So we had better resist any temptation to adopt such eugenic policies.

## CONCLUSION

I have argued that there exists a derived negative right to procreative freedom, including a right to IVF and to the exercise of selective techniques such as PGD. This is an extensive freedom, including not only the right to the exercise of a responsible parenthood, but also, in rare cases, to wrong decisions. It includes also a right for less than perfect parents to the use of IVF, and for IVF doctors to assist them, if they want and can agree about the terms.

The reason that we must accept a right to morally speaking wrong decisions has to do with the fact that it is necessary to stay clear of the kind of eugenics we are familiar with from the recent past. Society should not hold any opinion about what sort of people there should be.

## Footnotes

i The idea has also been defended by Blackorby and colleagues,^10^ and by Broome.^11^ For an introduction to the discussion see Arrhenius *et al.*^12^
